# Phenomenology of Mood Disorders in Children and Adolescents

**DOI:** 10.1192/bjo.2024.183

**Published:** 2024-08-01

**Authors:** Poornima Khadanga, Sherina Moktan, Eesha Sharma, Kommu John Vijaysagar

**Affiliations:** 1 Northampton, Northampton, United Kingdom; 2 Patan Mental Hospital, Kathmandu, Nepal; 3 National Institute of Mental Health and Neurosciences, Bengaluru, India

## Abstract

**Aims:**

Psychiatrists frequently diagnose mood disorders in children. However, there is a limited understanding of clinical history and phenomenology of mood disorders in these phases of lifespan, and phenomenological variations in those with and without neurodevelopmental disorders (NDD). The primary objective of the study was to study, comparatively, the phenomenology in children clinically diagnosed with mania, depression and mixed affective disorder. The second objective was to study the phenomenological differences in diagnosed cases of mood disorder children with and without neurodevelopmental disorders.

**Methods:**

We conducted a semi-qualitative study of the clinical history and phenomenology in 120 children recruited from a tertiary care child and adolescent psychiatry service. Children with current diagnosis of depression, mania or mixed affective state, age less than 18 years, and appropriate consent/assent were included. Children with comorbid neurological disorders, any underlying organicity, or those currently in remission from their mood episode were excluded. Descriptive summaries were calculated for socio-demographic, clinical and phenomenological data. Chi Square test was used to examine statistical differences in prevalence of various phenomena across the clinical diagnostic groups.

**Results:**

The most common clinical diagnosis was depression (58.3%) followed by mania (25.8%) and mixed affective state (15%). Irritable mood and emotional dysregulation were equally distributed among the three diagnostic groups. With a high prevalence of comorbid NDDs in the sample, we compared phenomena between groups with and without NDDs. In cases of depression, suicidal ideas and guilt feelings were expressed in 61% and 80% of these participants without comorbid NDD (n = 45) respectively, which was significantly high as compared with those with NDD (n = 67). The symptoms of disinhibition (78.9%), impulsivity (84.2%) and emotional dysregulation (10.5%) were more frequently seen in participants with neurodevelopmental disorder. Dissociative and obsessive phenomena were present in about a quarter of our study sample, similarly across mania, depression and mixed state.

**Conclusion:**

Mental status examination of mood disorders in children suggests considerable phenomenological overlap with irritable mood, emotional and behavioural dysregulation, dissociative symptoms, obsessive symptoms, sleep disturbances, nightmares and hyperarousal seen in mania, depression and mixed states. These phenomena may, therefore, not be suitable in differentiating these clinical diagnoses. Children with NDDs may report lesser cognitive phenomena of depression, and the clinician may have to rely on the affective and behavioural manifestations of depression in clinical decision-making.

